# Assessment of the influence of intrinsic environmental and geographical factors on the bacterial ecology of pit latrines‡

**DOI:** 10.1111/1751-7915.12334

**Published:** 2016-02-15

**Authors:** Belen Torondel, Jeroen H.J. Ensink, Ozan Gundogdu, Umer Zeeshan Ijaz, Julian Parkhill, Faraji Abdelahi, Viet‐Anh Nguyen, Steven Sudgen, Walter Gibson, Alan W. Walker, Christopher Quince

**Affiliations:** ^1^ Environmental Health GroupDepartment of Disease ControlLondon School of Hygiene and Tropical MedicineKeppel StreetLondonWC1E 7HTUK; ^2^ Pathogen Molecular Biology DepartmentFaculty of Infectious and Tropical DiseasesLondon School of Hygiene and Tropical MedicineKeppel StreetLondonWC1E 7HTUK; ^3^ School of EngineeringUniversity of GlasgowGlasgowG12 8LTUK; ^4^ Pathogen Genomics GroupWellcome Trust Sanger InstituteWellcome Trust Genome CampusHinxtonCB10 1SAUK; ^5^ Ifakara Health Instituteoff Mlabani PassageP.O. Box 53IfakaraTanzania; ^6^ Hanoi University of Civil Engineering55 Giai Phong RoadHanoiVietnam; ^7^ Bear Valley VenturesBraeside, Utkinton LaneCotebrookTarporleyCheshire CW6 0JHUK; ^8^ Microbiology GroupRowett Institute of Nutrition and HealthUniversity of AberdeenAberdeenAB21 9SBUK

## Abstract

Improving the rate and extent of faecal decomposition in basic forms of sanitation such as pit latrines would benefit around 1.7 billion users worldwide, but to do so requires a major advance in our understanding of the biology of these systems. As a critical first step, bacterial diversity and composition was studied in 30 latrines in Tanzania and Vietnam using pyrosequencing of 16S rRNA genes, and correlated with a number of intrinsic environmental factors such as pH, temperature, organic matter content/composition and geographical factors. Clear differences were observed at the operational taxonomic unit, family and phylum level in terms of richness and community composition between latrines in Tanzania and Vietnam. The results also clearly show that environmental variables, particularly substrate type and availability, can exert a strong structuring influence on bacterial communities in latrines from both countries. The origins and significance of these environmental differences are discussed. This work describes the bacterial ecology of pit latrines in combination with inherent latrine characteristics at an unprecedented level of detail. As such, it provides useful baseline information for future studies that aim to understand the factors that affect decomposition rates in pit latrines.

## Introduction

Annually, 2.4 million deaths could be prevented through improved hygiene, drinking water and sanitation (Bartram and Cairncross, 2010). Although great strides have been made, there are still 2.6 billion people worldwide without access to improved sanitation facilities (WHO/UNICEF, 2013), and for those trying to bring about improvements, the choice of technology is very limited. For the urban and rural poor the only available and affordable improved sanitation option is ‘on‐site sanitation’ such as pit latrines. A pit latrine consists of a dug hole, covered by a platform with a drop hole, and a superstructure in order to provide privacy to the users (Supplemental Fig. S1). Pit latrines can either be constructed simply in soil, or be lined with bricks in order to prevent the collapse of surrounding soil into the pit. One of the most important problems, particularly in high‐density urban areas, is that all pits, no matter what the initial volume, will eventually fill and must be emptied or replaced. Emptying or replacement can involve inconvenience, costs and health risks. Pit lifetimes, based on anecdotal evidence, are variable; some might fill up within 18 months while others have been reported to have a seemingly indefinite lifespan. Pit lifetime is likely to depend on a variety of factors, the most important of which are the number of users, the size of the pit, the degree to which the pit is drained and the degree to which the pit is used for disposal of other household wastes. Theoretically, a pit latrine in which the rate of breakdown is higher than the rate of filling should be achievable if the right microbes and environmental conditions are present.

There have been a limited number of studies that have reported on decomposition within pit latrines, and only anecdotal evidence into factors that can slow down or speed up decomposition (Couderc *et al*., 2008; Nwaneri *et al*., 2008). Commercial (bio)additives, often based on nutrients, enzymes or bacteria, claim increased decomposition rates, reduced odour and longer pit life spans (Foxon *et al*., 2008), though there is no scientific evidence that these products work, as was shown by a study conducted in South Africa (Buckley *et al*., 2008). Regardless, microbial communities will play an important role in organic matter degradation within pit latrines, though little is known about the microbial communities present in pit latrines and their association with faecal decomposition within the pit environment. In contrast, literature is available on the diversity of microbial communities in activated sludge wastewater treatment plants (Juretschko *et al*., 2002; Hoshino *et al*., 2006) and in biofilms in household toilets (McBain *et al*., 2003; Egert *et al*., 2010), where studies found a high prevalence of the phyla *Acidobacteria*, *Actinobacteria*, *Bacteroidetes*, *Planctomycetes* and *Proteobacteria*. However, very few of these studies have studied the relationship between microbial community structure and the degradation process.

The microbial communities present in a pit latrine likely represent a combination of those originating from human faeces and microbes from the environment, which might enter the pit latrine through the addition of household waste, or from surrounding soil or groundwater (McLellan *et al*., 2010). The human intestinal microbiota is composed of 10^13^–10^14^ microorganisms (Gill *et al*., 2006), is populated predominantly by strictly anaerobic bacteria from the phyla *Firmicutes*, *Bacteroidetes* and *Actinobacteria*, and includes a number of currently uncultured microbes (Eckburg *et al*., 2005; Zoetendal *et al*., 2008; Arumugam *et al*., 2011). Soil bacterial diversity is known to be immense (Dunbar *et al*., 2002; Tringe *et al*., 2005), with the number of distinct genomes present in a gram of soil ranging from 2000 to 18 000 (Torsvik *et al*., 1990; Sandaa *et al*., 1999). Members of the phyla *Proteobacteria* and *Acidobacteria* are the most abundant soil bacteria, and together with the *Actinobacteria*, *Verrucomicrobia*, *Bacteroidetes*, *Chloroflexi*, *Planctomycetes*, *Gemmatimonadetes* and *Firmicutes* phyla make up an average of 92% of the soil microbiota (Janssen, 2006). It is also known that the diversity of soil bacterial communities can be influenced by a wide range of biotic and abiotic factors, with pH playing an important role (Fierer and Jackson, 2006). The total number of bacteria found in groundwater ecosystems may vary by several orders of magnitude, between 10^2^ and 10^6^ cells per cm^3^ of ground water and between 10^4^ and 10^8^ cells per cm^3^ of sediment (Griebler and Lueders, 2009). Both cultivation‐dependent and cultivation‐independent surveys have revealed that groundwater communities are dominated by diverse heterotrophic *Proteobacteria*, *Actinobacteria*, *Firmicutes* and *Bacteroidetes*. Different studies have also frequently detected several uncultivated lineages from phyla such as *Acidobacteria, Chloroflexi*, *Verrucomicrobia* and *Nitrospirae* (Dojka *et al*., 1998; Rooney‐Varga *et al*., 1999; Feris *et al*., 2004), as well as representatives from phyla for which cultured representatives are totally unavailable (Macbeth *et al*., 2004; Connon *et al*., 2005).

The relative contribution of these two types of microbes (i.e. those present in human faeces, and those from the environment) on pit latrine community composition has yet to be determined. The work presented in this paper investigates the diversity and composition of bacterial communities found in pit latrines from different geographical regions, in tandem with physical and bio‐chemical characteristics (intrinsic environmental factors) of pit latrine material, in order to better understand the decomposition process.

## Results

### Alpha diversity

**Figure 1:**
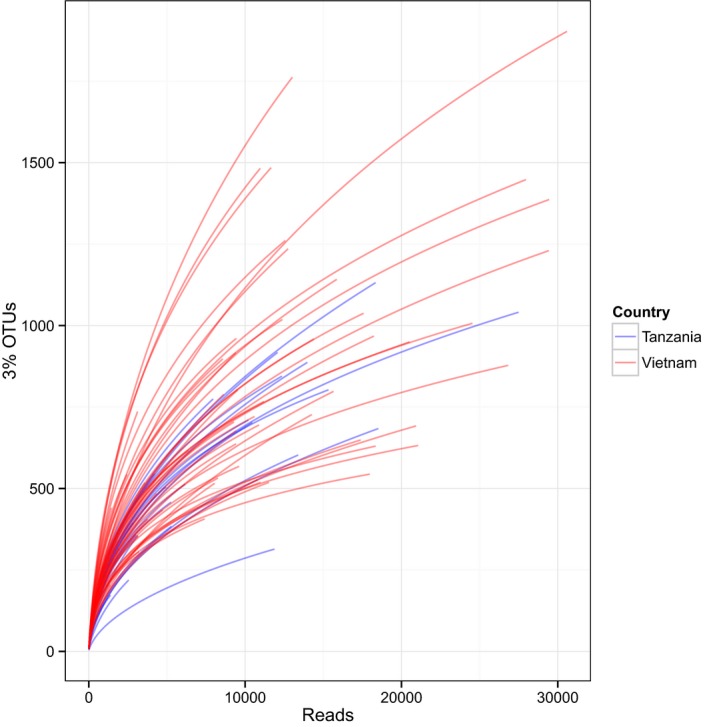
Richness: Alpha diversity rarefaction plots of 3% OTU richness for the latrines separated by country.

The observed diversities in the individual latrine samples were relatively high compared with the human gut (Turnbaugh *et al*., 2010). The number of 3% divergence (i.e. 97% similarity) operational taxonomic units (OTUs) in a sample can be used as an approximate proxy to species richness. We observed 3% OTU diversities that varied from 173 to 1903. Inevitably, sample size will impact the number of OTUs, but from the rarefaction curves given in Fig. 1, we observed that at least for the most deeply sequenced samples there was evidence that the curves were almost saturating.

To account for the impact of sequencing depth on richness, we used parametric methods to extrapolate to total diversities. These values (Table 1) and the observed OTU richness, both fall between those typically found in the human gut, a relatively low diversity environment, and soil, a highly diverse microbial community (Turnbaugh *et al*., 2010; Singh *et al*., 2014). Comparing the latrines from Vietnam and Tanzania, we observed a significantly lower per sample 3% OTU number in the Tanzanian latrines (mean = 530 versus 704, *P* < 0.01) (Table 1). This effect remained when samples were rarefied to 1000 sequences (mean 227 versus 247 *P* < 0.01) although it was not observed for the total diversity estimates suggesting that the Tanzanian latrines contain a longer tail of low abundance or rare OTUs. We also observed lower phyla and family numbers in the Tanzanian latrines per sample compared with Vietnamese latrines (mean 12.2 versus 13.9, *P* = 0.0047) and (mean 54 versus 81, *P* = 2.6e‐13) respectively.

**Table 1:** Summary of 16S rRNA gene sequence data and alpha diversities from Tanzania and Vietnam following noise removal. For the reads per sample, and diversities, the minimum, median and maximum values are given. Rarefactions were to a sample size of 1000 reads and were performed with the vegan rarefy function (Oksanen *et al*., 2012). Total diversity estimates were obtained by a parametric fit of the log‐normal distribution (Quince *et al*., 2008)

	Vietnam	Tanzania
Number of sequences	670374	213 898
Number of samples	55	24
Number of latrines	22	6
Reads per sample	1370 – 10900 – 30600	1059 – 7064 – 27 500
3% OTUs per sample**	205, 704, 1903	173, 529.5, 1132
3% OTUs rarefied**	158.3, 247.2, 428.9	75.0, 226.7, 273.8
3% OTUs total	1018, 3227, 151 972	1090, 16 012, 344 359
Families per sample***	52, 81, 122	30, 54, 81
Families rarefied***	41.17, 53.81, 75.01	19.81, 32.51, 49.30
Phyla per sample**	8, 14, 21	8, 12, 17
Phyla rarefied***	5.77, 10.04, 14.40	6.87, 9.10, 11.02

For the richness statistics t‐tests were performed to detect significant differences between the latrines from the two countries (* *P* < 0.05, ** *P* < 0.01, *** *P* < 0.001).

### Influence of intrinsic environmental parameters on 3% OTU richness

We also explored correlations between the different intrinsic environmental variables (physical and bio‐chemical factors measured in latrine contents to describe the prevailing latrine environmental conditions). More detailed descriptions are given in the *Methods* section characterizing the latrines (Supplementary Fig. S2). From this figure, we observed that total solids (TS) and pH are positively correlated with each other but are negatively related to all the other environmental variables, which are then mostly positively correlated with each other.

**Figure 2:**
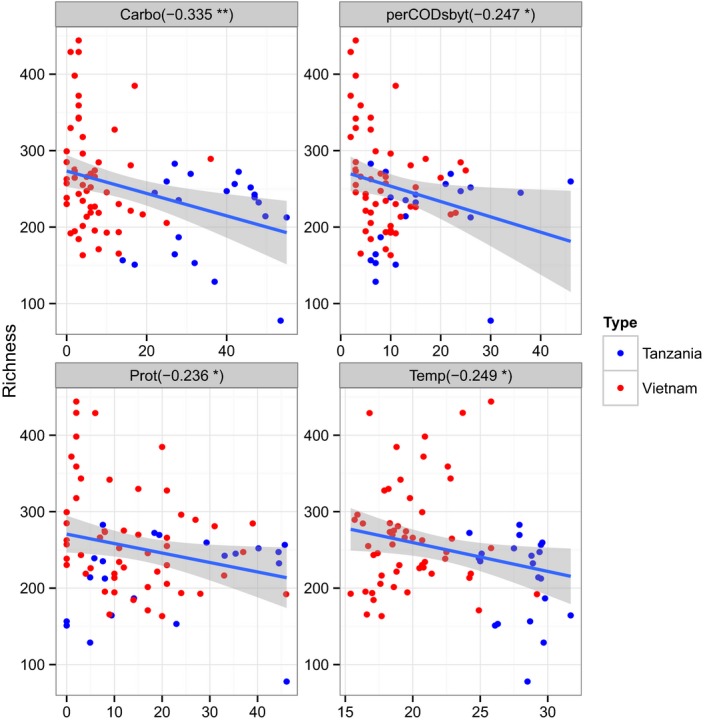
Impact of environmental parameters on 3% OTU richness. Each scatter plot shows a different environmental variable (x‐axis) versus rarefied 3% OTU number (y‐axis). Pearson's correlations are given and significance indicated (*P < 0.05, **P < 0.01, ***P < 0.001). Vietnamese samples are shown in red and Tanzanian in blue. (Carbo = carbohydrates, perCODsbyt = % of total COD converted to soluble COD, Prot = protein, Temp = temperature).

The correlations between these variables, and the rarefied 3% OTU richness are shown in Fig. 2. This figure shows that there was a highly significant negative correlation between percentage carbohydrate and OTU richness, and significant negative impacts of protein, perCODsbyt [% of total chemical oxygen demand (COD) converted to soluble COD] and temperature. These finding indicate that when there are more substrate resources available, there is a reduction in microbial diversity (OTU richness).

### Community comparisons

**Figure 3:**
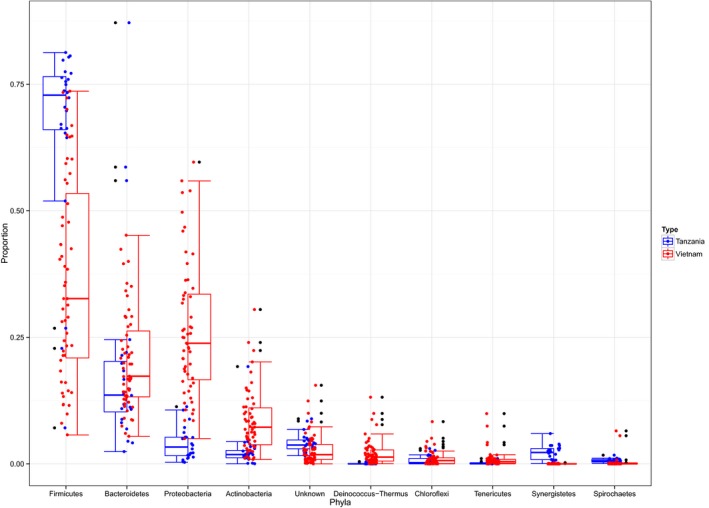
Mean phylum‐level proportion abundances in the Vietnamese and Tanzanian pit latrines. Top 10 most abundant out of a total of 30 observed phyla are shown.

The bar plot in Fig. 3 displays the proportions of the top 10 most abundant phyla in the Vietnamese and Tanzanian samples ordered by aggregate abundance. The most abundant phylum, comprising 37% and 66% of the latrine communities in Vietnam and Tanzania, respectively, was the *Firmicutes*, followed by the *Bacteroidetes*, *Proteobacteria* and *Actinobacteria* in order of decreasing abundance. There are clear differences between the proportional abundances of phyla between the two countries, and these differences are found to be highly significant at the whole community level from the permutational multivariate analysis of variance (*P* < 0.001), explaining 31% in the variance of the community structures. From fitting a Dirichlet‐multinomial model to the two countries separately (Supplementary Table S1), we can show that *Proteobacteria*, *Actinobacteria*, *Deinococcus‐Thermus* and *Verrucomicrobia* are all significantly more proportionally abundant in the Vietnamese latrines, whereas the *Firmicutes*, *Synergistetes* and *Spirochaetes* are more prominent in Tanzania.

**Figure 4:**
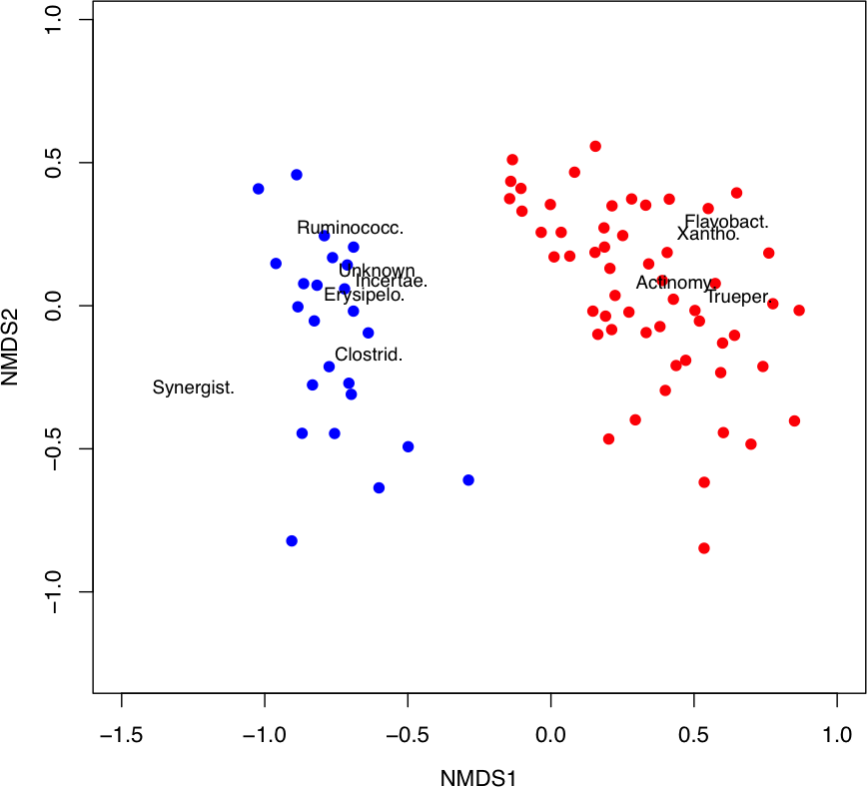
Non‐metric multidimensional scaling plot of family compositions for the Vietnamese (red) and Tanzanian samples (blue). The top 10 families that most differ in expected proportion under a Dirichlet‐multinomial model fit are biplotted (Supplementary Table S2). Clostrid = Clostridiaceae, Erysipelo. = Erysipelotrichaceae, Unknown = Unknown, Xantho = Xanthomonadaceae, Flavobact = Flavobacteriaceae, Trueper = Trueperaceae, Ruminococc. = Ruminococcaceae, Incertae. = Incertae Sedis XIV, Actinomy = Actinomycetales, Synergist. = Synergistetes.

These differences continue at the family level. The country that the latrine was derived from explains 14% of the variation in family‐level community structure (permutational multivariate analysis of variance *P* < 0.001). The Dirichlet‐multinomial model finds a large number of families that differ significantly in abundance between the countries (Supplementary Table S2). These results are summarized in Fig. 4 where a non‐metric multidimensional scaling (NMDS) plot of the samples is shown. The two countries clearly cluster into two separate groups. On this diagram, we also biplot the locations of the 10 families with the largest absolute difference in expected proportional abundance between the two countries. *Synergistaceae*, and the *Firmicutes* lineages *Clostridiaceae*, *Ruminococcaceae*, Incertae Sedis XI and *Erysipelotrichaceae* were associated with Tanzanian pits, while *Xanthomonadaceae*, *Actinomycetales*, *Flavobacteriaceae* and *Trueperaceae* were more likely to be found in Vietnamese pits. The impact of the country where the latrine was sampled on latrine community composition is also detected at the 3% OTU level, our proxy for species, and 11% of the variation in 3% OTU community structure is explained by the country of origin (*P* < 0.001).

**Figure 5:**
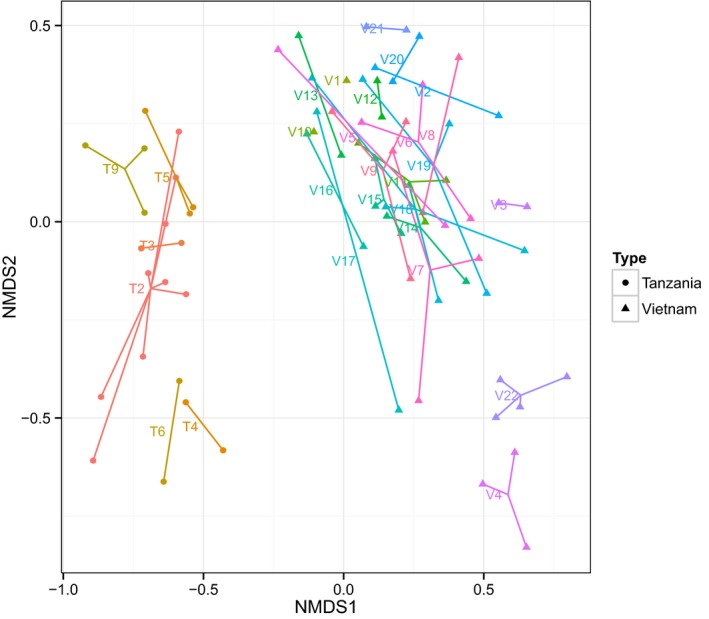
NMDS plot of 3% OTUs compositions for the Vietnamese and Tanzanian samples coloured and grouped by latrine identity (each colour represents one latrine).

In addition to the impact of geographical location described above, to explore the impact of both latrine identity (i.e. inter‐latrine variability, incorporating differences between distinct latrines) and the depth within latrines that the samples were taken from (intra‐latrine variability), on the community composition we performed a multivariate analysis of variance at the phylum, family and 3% OTU levels. At the phylum level, latrine identity had a further significant impact on community composition but depth did not (Supplementary Table S4). Similarly, at the family level (Supplementary Table S5), country of origin explained 22% of the variance but including latrine identity explained a further 47%, and both of these variables had highly significant *P*‐values (*P* = 0.001). Depth was significant (*P* = 0.009) but only explained 2% of the variance. The same was found for the 3% OTU compositions (Supplementary Table S6), with explained variances of 15%, 48% and 2% respectively. Figure 5, to graphically illustrate the impact of latrine identity, shows an NMDS plot of the 3% OTU compositions grouped by latrine.

These analyses establish that both latrine identity and country of origin have a major impact on community structure at all taxonomic levels, while depth within a given latrine has a lesser, but still significant, impact.

We next attempted to determine the extent to which this is due to differences in the local intrinsic environment of the latrines and the extent to which it may depend on other factors, such as the gut microbiota or diets of the individuals using the latrines, or indeed the local soil microbiota by including the measured intrinsic environmental variables in the multivariate analysis of variance. The results are shown for family composition in Supplementary Table S7. Many of the environmental variables are shown to be significantly correlated with the latrine community structure. In fact, only those variables associated with COD fail to have a significant impact [CODt, CODs, (%CODs/CODt)], suggesting that the amount of resources available is less important than the type of resources (e.g. % carbohydrate) or abiotic environment (pH and temperature) in structuring the community. After incorporating the intrinsic environmental data, latrine identity still explains a large amount of variation (33%), whereas the country, while still significant, is less important (6%). Depth is no longer a significant driver of community structure. This implies that all the effect of depth was due to correlation with the environment and that most of the difference between Tanzania and Vietnam is driven by the intrinsic environmental factors. However, the remaining highly significant impact of latrine identity implies a role for local factors beyond the intrinsic environment or for environmental factors that we did not measure.

There are clearly large differences between the latrine microbiota of the two countries. To investigate whether intrinsic environment controls microbiota within a country, we separated the samples by country of origin and performed permutational multivariate analysis of variance on the family level community composition for each separately. The results are given for Vietnam and Tanzania in Supplementary Tables S8 and S9 respectively. In Vietnam more significant results were found with TS, volatile solids (VS), volatile fatty acids (VFA) and protein all impacting the pit latrine microbiota, whereas only TS and %CODs/CODt were significant in Tanzanian latrines.

**Figure 6:**
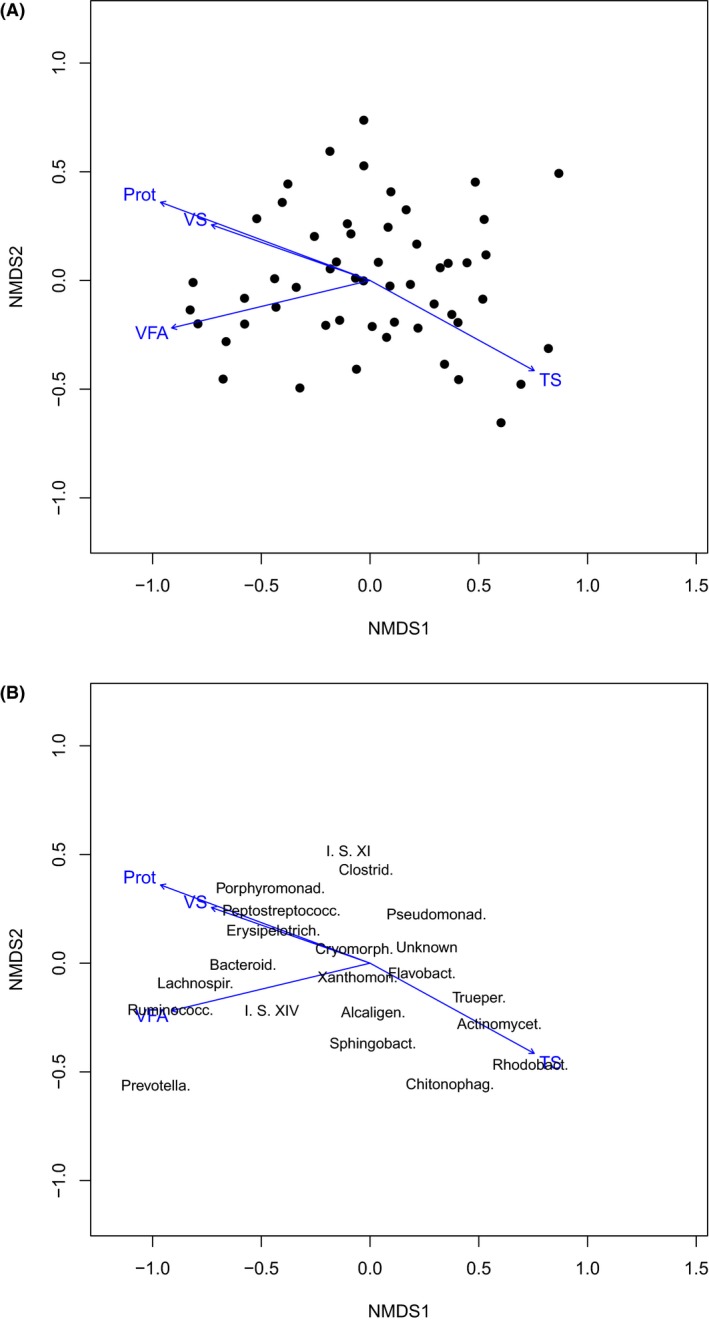
Non‐metric multidimensional scaling of family compositions of Vietnamese latrine samples with the four environmental variables judged significant in Supplementary Table S7 (total solids = TS, volatile solids = VS, volatile fatty acids = VFA and protein = Prot) plotted using the envfit function of vegan. The left panel gives the locations of the latrine samples and the right panel the 21 families with an average abundance of greater than 1%.

In order to explore the relationships within Vietnam in more detail, we generated a NMDS plots for both the samples and all the families with an average abundance of greater than 1% (Fig. 6). Onto these we plotted the direction of change in the four significant environmental variables. From this, we observed that an environmental gradient exists associated with VS, VFA and protein, and which is negatively associated with TS. This gradient controls the community structure, shifting it from environmental associated families (such as *Actinomycetales* and *Rhodobacteraceae*) to gut associated organisms (such as *Bacteroidaceae*, *Prevotellaceae* and *Lachnospiraceae*). The nature of this gradient is given in more detail, complete with estimated contours, in Supplementary Fig. S3.

## Discussion

Microbial populations within pit latrines are likely derived from a combination of species originating from human faeces and microbes from the environment, which might enter the pit latrine through the addition of household waste, or from surrounding soil or groundwater.

When faecal bacterial communities leave the gut, their survival and persistence will depend on their ability to respond to changing environmental surroundings. This ability is governed by cellular physiology, stress response mechanisms and the physical properties of the bacterium. Examples of these include adaptations to varying availability of nutrients e.g. low iron, and variation in atmospheric oxygen percentage, and the ability to survive by changing physical properties such as spore formation (Kim *et al*., 2011; Leffler and Lamont, 2012). The selective persistence of specific subpopulations from faecal‐associated microbiota will have a significant influence on microbial population structures outside of the human body, including shifts in composition of high and low abundance taxa (McLellan *et al*., 2010).

A small number of studies have provided information about the composition of untreated sewage microbial communities in developed countries (McLellan *et al*., 2010; VandeWalle *et al*., 2012; Shanks *et al*., 2013), and these have showed that the sewage profile includes a core human faecal signature made up of several abundant taxonomic groups within the *Firmicutes*, *Bacteroidetes*, *Actinobacteria* and *Proteobacteria* phyla (Shanks *et al*., 2013). In our study, we found the same predominant phyla in pit latrines from both Tanzania and Vietnam. However, there were significant differences between Tanzanian and Vietnamese pit latrines. Many of the significantly more proportionally abundant families in Tanzanian pit latrines (60.45% versus 32.15%) were part of the *Firmicutes* phylum, and are common inhabitants of the human gut (Gill *et al*., 2006) e.g. *Clostridiaceae*, *Ruminococcaceae* and *Erysipelotrichaceae*. The phylum *Spirochaetes* was also found to be significantly more proportionally abundant in the Tanzanian pit latrines, and organisms from this phylum have previously been shown to be important components of the gut microbiota in individuals from rural, less developed, regions (Cooper *et al*., 2013; Schnorr *et al*., 2014). Thus, a larger proportion of the dominant microbiota in Tanzanian pit latrines appears to be derived from faeces than in the counterpart latrines in Vietnam. This variation is likely to occur in large part because of intrinsic environmental factors within the pit latrines. Interestingly, users of Vietnamese pit latrines often spread ash and lime on top of the latrines, which results in elevated pH levels, among other changes in environmental conditions, and this may affect the survivability of the faecal species deposited in Vietnam pit latrines. The presence of a number of alkalinophilic *Proteobacteria* species in Vietnamese latrines, and their absence from Tanzanian latrines, adds further evidence to suggest that alterations in pH levels due to ash and lime use are important differentiators of microbial community composition between the two countries.

The phylum *Synergistetes* was also found to be present at significantly higher proportional abundances in the Tanzanian pit latrines compared with Vietnam pit latrines. *Synergistetes* are anaerobic bacteria and are known for inhabiting many anaerobic environments, e.g. animal gastrointestinal tracts and soil (Jumas‐Bilak *et al*., 2007; Vartoukian *et al*., 2007). Interestingly, certain species from this phylum have been identified to play an important role in the degradation of sludge for production of biogas in anaerobic digesters (Riviere *et al*., 2009).

At the phylum level, *Actinobacteria* were found to be significantly more proportionally abundant in Vietnamese pit latrines when compared with Tanzanian pit latrines. They are one of the most abundant bacterial phyla found in soil, freshwater and marine environments, where they have key roles in the decomposition of organic material such as cellulose and chitin, and are important in organic matter turnover and the carbon cycle. Actinomycetes isolated from soil and related substrates show primary biodegradative activity, secreting a range of extracellular enzymes and exhibiting the capacity to metabolize recalcitrant molecules (McCarthy and Williams, 1992).

At the familylevel, one taxon that showed significantly higher proportional abundances in Vietnamese pit latrines compared with those in Tanzania was the *Xanthomonadaceae*, typically characterized as environmental organisms which are found in soil and water, as well as plant tissues (Arrieta‐Ortiz *et al*., 2013; Hui *et al*., 2014; Kelly *et al*., 2014). Recent studies have shown degradation properties for organisms from this family (Cea *et al*., 2010; Liu *et al*., 2011). *Trueperaceae* were also found to be more proportionally abundant in Vietnam compared with Tanzania's. The *Trueperaceae* family belongs to the phylum Deinococcus‐Thermus, and some researchers have reported the presence of this family in extreme environments (Albuquerque *et al*., 2005). Some members of the Deinococcus‐Thermus phylum are inherently resistant to environmental hazards (Griffiths and Gupta, 2007) and so one possible reason for there being more in the Vietnam pit latrines is due to the addition of lime and ash.

In summary, Vietnamese pits contain more halophilic/alkaliniphilic organisms, while Tanzanian pits appear to have more cellulose degrading, gut‐dwelling organisms. There are a number of factors, such as differences in gut microbiota composition, diet, anal cleansing habits (Tanzanians use water for anal cleansing while Vietnamese use paper), and latrine maintenance approaches like different emptying patterns that can potentially explain the differences in pit latrine microbiota composition observed between the two countries and between each latrine. Tanzanian users have a predominantly vegetarian diet compared with the Vietnamese population, which may explain why in Tanzania we found bacteria groups associated with cellulose degradation.

Taken together, the divergent nature of our results, and range of both obligatory anaerobic and aerobic species detected, indicates that there are both aerobic and anaerobic degradation processes occurring within the pit latrine environment, and that these processes will likely differ according to the intrinsic pit environmental conditions. Our results also demonstrate that each latrine has a distinct bacterial community composition, and that differences in composition *between* latrines are far greater than differences *within* latrines. One potential explanation for this is that the rate of growth and change in the latrine microbiota may slow as resources become depleted. One limitation of 16S rRNA gene sequencing is that it is unable to distinguish between live and dead/inactive microbes, potentially limiting our ability to detect the level of growth and activity of the microbial communities present at different depths within each latrine. Nonetheless, latrine identity and country of origin were both shown to have a major impact on community structure at all taxonomic levels. However, the local intrinsic environment of the latrines has a greater influence on the community structure. Only variables associated with COD failed to have a significant impact, suggesting that total available resources are less important than the type of resources or abiotic environment in structuring the community. Previous work (Turnbaugh *et al*., 2008; Le Chatelier *et al*., 2013) in related environments such as the human gut suggest that increased fibre consumption is correlated with increased microbial diversity but the opposite pattern can occur when polysaccharides are provided in excess, possibly due to domination of the microbial community by a small number of specific lineages that can utilize particular substrates (Walker *et al*., 2010).

## Conclusions

In this study, we carried out the first in‐depth, DNA sequence‐based characterization of pit latrine microbiota, and revealed which environmental factors can exert strong influences over bacterial community assembly and structure. The country where the latrines are from, and the individual intrinsic characteristics of each latrine, may have major impacts on community structure at all taxonomic levels. Available energy sources could play a crucial role in influencing pit latrine bacterial communities; however, this study indicates that the amount of resources is likely to play a less important role than the type of resources and the abiotic environment. The insights gained from this study provide useful baseline information for future attempts to alter the intrinsic microbial ecology and environmental conditions of pit latrines, with the eventual goal of optimizing decomposition rates.

## Experimental procedures

### Study area

The study was conducted in two countries, Tanzania and Vietnam, which provided access to contrasting and diverse non‐piped, on‐site pit‐based sanitation systems. In Tanzania, latrines were selected in villages close to the rural town of Ifakara, while in Vietnam, a community was selected close to the capital Hanoi. We selected these two countries to get a contrasting set of different pit latrines systems, and we also selected the sites that were close to a laboratory facility in order to perform all the analysis.

#### Tanzania

The villages of Sululu and Signali, where the latrines were selected, are situated in the Kilombero river valley, close to the town of Ifakara in the Morogoro region of southern‐central Tanzania. Most inhabitants are involved in subsistence agriculture, and diets are predominantly vegetarian. Households are dispersed within the village, with latrines placed within the compound. Pit latrines were constructed in soil and were 2 m deep on average (Supplementary Fig. S1). Tanzania has a tropical climate; typically it experiences a short rainy season from November to December and longer heavier rains from April to May. Average daily temperatures range from 19°C to 32°C.

#### Vietnam

In Vietnam, the neighbouring communes of Hoang Tay and Nhat Tan, Ha Nam, province (roughly 60 km south of central Hanoi) were selected for this study. The area is predominantly agricultural with intensive rice cultivation and animal husbandry (pigs, chickens and ducks). Pits in Vietnam are raised in order to allow excreta collection for use in agriculture, and pit volume is much smaller than that typically found in Tanzania, with the vaults 1 metre deep at maximum (Supplementary Fig. S1). Many households separate urine from faeces and paper is used for anal cleansing. The use of ash or lime to reduce odours is widely practised. Diet in Vietnam is diverse though tends to consist of rice, noodles, a variety of green vegetables and meat or poultry. Average daily temperatures range from 3°C to 38°C.

### Latrine selection

A total of 30 latrines were selected for this study. Latrines were selected based on the number of users, design characteristics, such as presence or absence of a roof, and materials used for construction. Eight latrines were selected in Tanzania, while 22 were selected in Vietnam. Characteristics of selected latrines from both countries are presented in Table 2

**Table 2:** Characteristics of the 30 selected latrines in Tanzania and Vietnam

	Tanzania (Ifakara)	Vietnam (Peri‐urban Hanoi)
Latrine type (%)		
Single vault	100	75
Double vault	0	25
Latrine ownership type (%)		
Family	50	100
Shared	0	0
Communal	50	0
N° Users [Mean (min–max)]	10.75 (5–20)	4.5 (2–7)
Depth [Mean (min–max)] (m)	1.45 (1.22–3.05)	0.65(0.41–0.96)
Latrine structure (%)		
Roof (Roof/No roof)	33.3/66.6	100/0
Wall (grass–bricks)	50/50	0/100
Vault (lined–unlined)	33.3/66.6	80/20
Slab type (%)		
Soil/ Cement or brick	50/50	0/100
Soil type	Sandy/loam	Clay/loam
Climate		
Rainfall average (min‐max) (mm)	120 (77–156)	158 (141–183)
Temperature average (min–max) (°C)	25 (19–32)	23.3 (3.2–38.2)
Anal cleansing	Water	Paper + water
Diet	Predominantly vegetarian	Predominantly omnivores
Excreta management	Disposal	Use in agriculture
Urine (%)		
Disposed in pit/ Urine separators	100/0	60/40

a. Data from the last 10years.

b. Monthly average.

### Sample collection and analysis

Approximately 200 g of material was collected at 20 cm intervals from top to bottom in each pit latrine. Latrines in Tanzania were much deeper and as a result more samples could be collected from each latrine. Depending on the consistency of the material in the pit, samples were collected with a standard soil auger (Eijkelkamp, Giesbeek, the Netherlands), or with a sterile 150 ml plastic container attached to the soil auger. In cases where the liquid layer was deeper than 20 cm, samples were collected with a sampler that was specifically designed for this project, which allowed us to collect samples from deep layers. The sampler consisted of a sampling tube that covers a gate with a spring‐loaded fin device (Supplementary Fig. S4). The sampler was inserted into the latrine and, once the required layer was reached, the device opened and a lever was used for collecting a small amount of sample. *In‐situ* temperature and pH measurements were taken with a hand‐held meter (HI 991003, Hanna Instruments, USA). Pit latrine samples were collected in sterile containers and transported in a cool box for further analysis on the same day as collection. Samples were analysed for TS and moisture content as indicators of the amount of inorganic and water content in a sample respectively. Total and soluble COD and VS were measured to give a gross and indirect indication of the organic matter content present in each sample, and measurements of carbohydrate and protein content provided information about the type of organic substrates present in each sample. Volatile fatty acids are important intermediates and metabolites in biological processes, and their presence in a sample matrix is often indicative of bacterial activity. Ammonia and total phosphate were also measured in each sample as their concentrations are determined by microbiological processes. All these tests were selected to describe the prevailing latrine environmental conditions, which we hereafter refer to as intrinsic environmental factors.

Samples were homogenized by using a Homogeniser pack (Powergen 500, Fisher, UK) following which 1 g was diluted in 20 ml of _dd_H_2_O. After homogenization and dilution, the mixture was passed through a 0.45 μm filter. Samples were analysed using HACH‐Lange test kits and methods (HACH, 2012); for total and soluble COD using the dichromate method; for Ammonia using the Salicylate method; for VFA the TNT plus method; and for total phosphate by using a heat block (LT20, HACH‐Lange, Loveland, USA) and spectrophotometer (DR2800, HACH‐Lange, Loveland, USA). Moisture, total and VS content of the samples were measured using standard waste analysis protocols (APHA‐AWWA‐WEF, 2005), with samples dried at 103–105°C for total solids and moisture content, and ignited at 550°C for volatile solids. Total protein levels in each sample were measured using the Lowry assay method (Lowry *et al*., 1951), while carbohydrate content was assessed by the phenol‐sulphuric acid technique (Masuko *et al*., 2005).

Samples for DNA sequence analysis were kept at −80°C until DNA extraction was performed. Deoxyribonucleic acid was extracted from the samples following the kit protocol for the FastDNA SPIN kit for soil (MP Biomedicals, Santa Ana, USA) in conjunction with a FastPrep‐24 bead‐beading instrument (MP Biomedicals, Santa Ana, USA).

### 454 pyrosequencing

Primers 338F and 926R, targeted towards variable regions 3 to 5 of the bacterial 16S rRNA gene, were used to generate amplicons from each sample. Each 926R primer contained a unique 12‐mer barcode sequence, plus the standard 454 Lib‐L kit ‘A’ adaptor (shown in bold), in the following configuration: 926R primers 5′‐**CCATCTCATCCCTGCGTGTCTCCGACTCAG**‐barcode‐CCGTCAATTCMTTTRAGT‐3′. The 338F primer contained the 454 Lib‐L kit ‘B’ adaptor (bold) followed by the 16S rRNA gene primer: 338F – 5′‐**CCTATCCCCTGTGTGCCTTGGCAGTCTCAG**ACTCCTACGGGAGGCAGCAG‐3′. Polymerase chain reaction (PCR) amplification of 16S rRNA genes from the extracted DNA involved initial denaturation at 94°C for 2 min; 20 cycles of denaturation (30 s at 94°C), annealing (30 s at 53°C), extension (68°C for 2 min). Amplification was performed using Accuprime Taq DNA polymerase from Life Technologies (Paisley, UK) using manufacturer's instructions. Polymerase chain reaction amplicons were generated in quadruplicate for each sample and then pooled. Amplicon concentrations were checked for each sample using a Qubit from Life Technologies (Carlsbad, CA, USA) and then added in equimolar amounts to a mastermix for sequencing. Polymerase chain reaction products were cleaned using the Wizard PCR product purification kit (Promega, Fitchburg, WI, USA) and were then pyrosequenced at the Wellcome Trust Sanger Institute using the Lib‐L kit on the 454 GS FLX Titanium System (Roche, Branford, CT, USA).

### Quality trimming and taxonomic assignments

The sequence data was processed using the AmpliconNoise pipeline (Quince *et al*., 2011), which uses the pattern of light intensities (or flow gram) associated with each read (Quince *et al*., 2009). The samples were de‐multiplexed by using their barcodes, requiring exact matches to both barcode and primer. The flow grams were then filtered and trimmed based on the identification of low quality signals (Quince *et al*., 2009). The filtered flow grams were clustered to remove errors and converted into sequences using the PyroNoise algorithm. The sequences had barcodes and degenerate primers removed prior to trimming at 400 bp. They were then further clustered by SeqNoise to remove PCR single base errors. Finally, the Perseus algorithm was used to identify chimeras (Quince *et al*., 2011).

### OTU clustering and taxonomic classification

The denoized sequences were classified using the standalone RDP classifier (Wang *et al*., 2007). From this, taxa frequencies at five different levels: phylum, class, order, family and genus; were calculated. In addition, a non‐supervised approach was used, OTUs being generated following pair‐wise Needleman‐Wunsch alignment and hierarchical clustering with an average linkage algorithm. OTUs were generated at 3% sequence cut‐off.

### Analysis of OTU richness and diversity

Laboratory/reagent contamination was monitored through comparison with a ‘blank’ DNA extraction sample, containing just pure water that was then PCR amplified using a unique barcoded primer and sequenced. This blank was dominated by previously described laboratory contaminants (Salter *et al*., 2014) and formed an outlier on OTU derived NMDS plots. Three Tanzanian samples had a similar composition to this blank, and clustered with it on the NMDS plots, so were removed from further analysis. The OTUs in these samples were only minor components of the non‐contaminated samples. We could be confident therefore that none of the other samples were significantly impacted by contamination which probably derived from improper handling of low DNA samples in Tanzania. Any samples with less than 1000 reads were also removed. Following the noise removal procedure outlined above nearly 1 million reads remained in total, with six of the eight original Tanzanian latrines represented, and all 22 Vietnamese latrines (Table 2). For all these samples, rarefaction curves for 3% OTUs were calculated to determine the extent to which these samples had been sampled (Fig. 1). The number of 3% OTUs, families and phyla observed in the Vietnamese and Tanzania latrines were also calculated and compared using *t*‐tests (Table 2). These quantities were rarefied to a common sample size of 1000 reads to account for the impact of sampling depth using the vegan rarefy function (Oksanen *et al*., 2012). Estimates of total diversity were also obtained using the parametric method described in Quince and colleagues (2008) (Table 2).

### Visualization of community structure using NMDS

Two dimensional visualizations of the whole community structure was performed using NMDS with Bray–Curtis distances at phylum, family and 3% OTU levels.

### Multivariate analysis of microbial community structure

To determine if environmental variables, and the categorical variables country and depth had a significant impact on latrine community structure, we used permutational multivariate analysis of variance. This is a distance‐based alternative to traditional multivariate analysis of variance (MANOVA) allowing the variance in a multivariate data set to be partitioned between both continuous and categorical variables (Anderson, 2001). We used the implementation of permutational MANOVA in the Adonis function of the vegan R package. In addition, to determine precisely how the community structure differed between categorical variables we fitted Dirichlet‐multinomial models to the community compositions to each category separately (Holmes *et al*., 2012). This allowed determination of differences in mean expected taxa abundances and uncertainties in those predictions.

## Author contributions

BT, WG, JE, AW, CQ, JP contributed to the study design. BT managed the study. BT, JE, SS, FA and VA performed the sample collection and analysis test on the field laboratories. BT and OG performed the DNA extraction and PCR. AW and JP performed the 454‐sequencing at the Wellcome Trust Sanger Institute. CQ and UZ performed the statistical analysis. BT and CQ drafted the paper. All authors contributed to redrafting. The authors wish to thank Paul Scott, Richard Rance and members of the Wellcome Trust Sanger Institute's 454 sequencing team for generating 16S rRNA gene data.

## Conflict of Interest

None declared.

## Supporting information


**Fig. S1.** Latrine examples from Tanzania and Vietnam.
**Fig. S2.** Correlations between environmental parameters. Each parameter is correlated with all the other ones by Pearson correlation. (VS = volatile solids, CODt = total chemical oxygen demand, CODs = soluble chemical oxygen demand, VFA = volatile fatty acids, Prot = protein, perCODsbyt = percentage of CODt converted into CODs, Carbo = carbohydrates, Temp = temperature, TS = total solids, pH).
**Fig. S3.** Non‐metric multidimensional scaling of family compositions of Vietnamese latrine samples with bubble plots and gradients for the four environmental variables judged significant in Supplementary Table S7 (total solids = TS, volatile solids = VS, volatile fatty acids = VFA and Prot = protein) plotted using the envirosurf function of vegan. The size of the bubble indicates the value of the environmental variable.
**Fig. S4.** Sampler device used to sample deep latrines with more liquid consistency material. (https://www.youtube.com/watch?v=q5JDu0emYxk).
**Table S1.** Percentage relative abundance of the 30 phyla in the Dirichlet means in the 55 Vietnamese samples and 24 Tanzanian samples. The upper and lower 95% credible intervals are also given. These are calculated as the maximum posterior estimate (MPE) minus/plus two standard deviations as calculated from the inverse Hessian. Phyla are ranked in order of their contribution to the total mean difference between the two means. This is the second from last column in the table. The total difference was 37%. The cumulative fraction of this difference accounted for by each family is given in the last column of the table.*Indicates those phyla that differ significantly between groups in that their confidence intervals do not overlap. Phyla that were more proportionally abundant in Tanzanian latrines are highlighted in blue; those that were more proportionally abundant in Vietnamese latrines are highlighted in red.
**Table S2.** Percentage relative abundance of the first 30 out of 180 families in the Dirichlet means in the 55 Vietnamese samples and 24 Tanzanian samples. The upper and lower 95% credible intervals are also given. These are calculated as the maximum posterior estimate (MPE) minus/plus two standard deviations as calculated from the inverse Hessian. Families are ranked in order of their contribution to the total mean difference between the two means. This is the second from last column in the table. The total difference was 37%. The cumulative fraction of this difference accounted for by each family is given in the last column of the table.*Indicates those families that differ significantly between groups in that their confidence intervals do not overlap. Families that were more proportionally abundant in Tanzanian latrines are highlighted in blue; those that were more proportionally abundant in Vietnamese latrines are highlighted in red.
**Table S3.** Percentage relative abundance of the first 10 out of 12,335 3% OTUs in the Dirichlet means in the 55 Vietnamese samples and 24 Tanzanian samples. The upper and lower 95% credible intervals are also given. These are calculated as the maximum posterior estimate (MPE) minus/plus two standard deviations as calculated from the inverse Hessian. OTUs are ranked in order of their contribution to the total mean difference between the two means. This is the second from last column in the table. The total difference was 129%. The cumulative fraction of this difference accounted for by each family is given in the last column of the table.*Indicates those families that differ significantly between groups in that their confidence intervals do not overlap. OTUs that were more proportionally abundant in Tanzanian latrines are highlighted in blue; those that were more proportionally abundant in Vietnamese latrines are highlighted in red.
**Table S4.** Permutational multivariate analysis of variance using Bray‐Curtis distances for the phylum latrine composition data as a function of country of origin and latrine identity (adonis function vegan – Oksanen *et al*., 2012). Signif. codes: ***< 0.001, **< 0.01, *< 0.05, < 0.1.
**Table S5.** Permutational multivariate analysis of variance using Bray–Curtis distances for the family latrine composition data as a function of country of origin and latrine identity (adonis function vegan – Oksanen *et al*., 2012). Signif. codes: ***< 0.001, **< 0.01, *< 0.05, < 0.1.
**Table S6.** Permutational multivariate analysis of variance using Bray‐Curtis distances for the 3% OTU latrine composition data as a function of country of origin and latrine identity (adonis function vegan – Oksanen *et al*., 2012). Signif. codes: ***< 0.001, **< 0.01, *< 0.05, < 0.1.
**Table S7.** Permutational multivariate analysis of variance using Bray‐Curtis distances for latrine composition family data as a function of intrinsic environmental variables, country of origin and latrine identity (adonis function vegan – Oksanen *et al*., 2012). Signif. codes: ***< 0.001, **< 0.01, *< 0.05, < 0.1.
**Table S8.** Permutational multivariate analysis of variance using Bray–Curtis distances for Vietnam latrine microbial composition family data as a function of intrinsic environmental variables (adonis function vegan – Oksanen *et al*., 2012). Signif. codes: ***< 0.001, **< 0.01, *< 0.05, < 0.1.
**Table S9.** Permutational multivariate analysis of variance using Bray–Curtis distances for Tanzanian latrine microbial composition family data as a function of intrinsic environmental variables (adonis function vegan – Oksanen *et al*., 2012). Signif. codes: ***< 0.001, **< 0.01, *< 0.05, < 0.1.
