# Supplementary material for: Assessment of the influence of intrinsic environmental and geographical factors on the bacterial ecology of pit latrines
Source: Microb Biotechnol. 2016 Feb 15;9(2):209–23. doi: 10.1111/1751-7915.12334 (PMC4767293; doi:10.1111/1751-7915.12334)
Supplement: Supplementary file 1 — Fig. S1. Latrine examples from Tanzania and Vietnam. Fig. S2. Correlations between environmental parameters. Each parameter is correlated with all the other ones by Pearson correlation. (VS = volatile solids, CODt = total chemical oxygen demand, CODs = soluble chemical oxygen demand, VFA = volatile fatty acids, Prot = protein, perCODsbyt = percentage of CODt converted into CODs, Carbo = carbohydrates, Temp = temperature, TS = total solids, pH). Fig. S3. Non‐metric multidimensional scaling of family compositions of Vietnamese latrine samples with bubble plots and gradients for the four environmental variables judged significant in Supplementary Table S7 (total solids = TS, volatile solids = VS, volatile fatty acids = VFA and Prot = protein) plotted using the envirosurf function of vegan. The size of the bubble indicates the value of the environmental variable. Fig. S4. Sampler device used to sample deep latrines with more liquid consistency material. (https://www.youtube.com/watch?v=q5JDu0emYxk). Table S1. Percentage relative abundance of the 30 phyla in the Dirichlet means in the 55 Vietnamese samples and 24 Tanzanian samples. The upper and lower 95% credible intervals are also given. These are calculated as the maximum posterior estimate (MPE) minus/plus two standard deviations as calculated from the inverse Hessian. Phyla are ranked in order of their contribution to the total mean difference between the two means. This is the second from last column in the table. The total difference was 37%. The cumulative fraction of this difference accounted for by each family is given in the last column of the table.*Indicates those phyla that differ significantly between groups in that their confidence intervals do not overlap. Phyla that were more proportionally abundant in Tanzanian latrines are highlighted in blue; those that were more proportionally abundant in Vietnamese latrines are highlighted in red. Table S2. Percentage relative abundance of the first 30 out of 180 f [file MBT2-9-209-s001.zip › Supplementary material.docx]

**Supplementary Figure 1:** Latrine examples from Tanzania and Vietnam.

Tanzanian Latrine


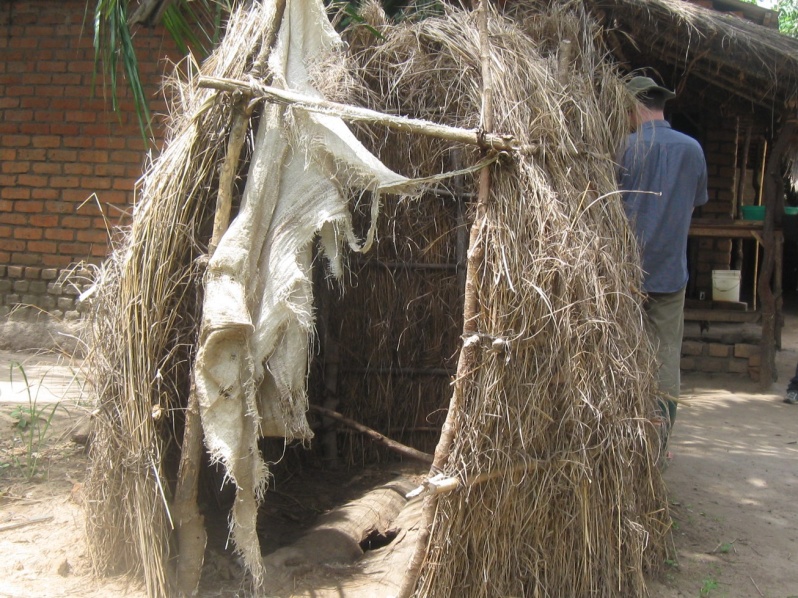


Vietnamese latrine


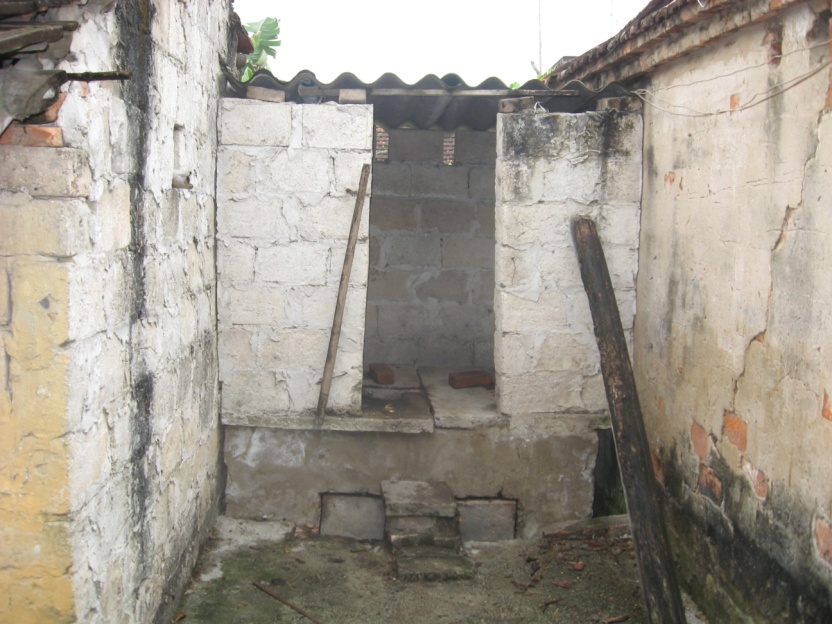


**Supplementary Figure 2:**

**
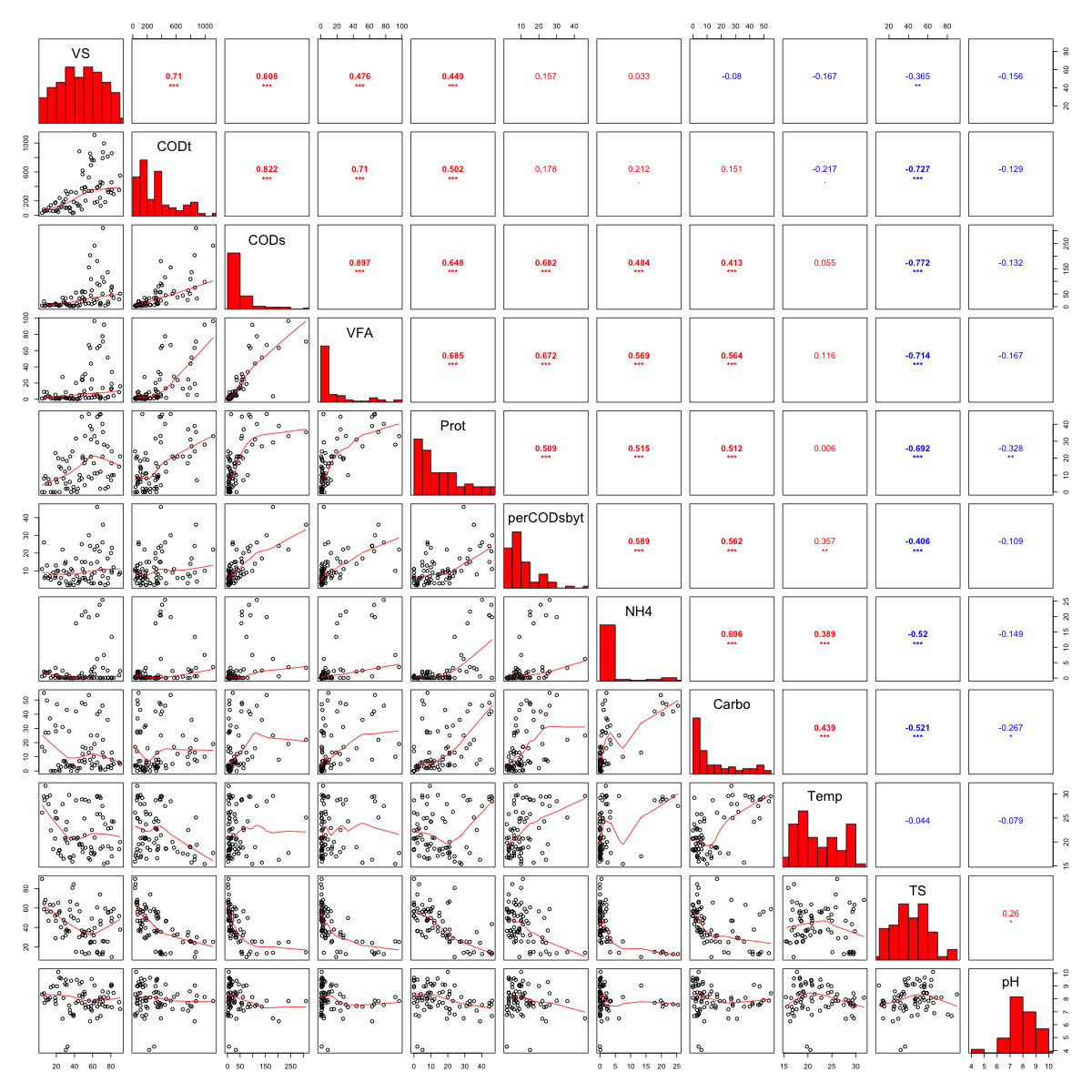
**

**Supplementary Figure 2:** Correlations between environmental parameters. Each parameter is correlated with all the other ones by Pearson correlation. (VS=Volatile solids, CODt=Total Chemical Oxygen Demand, CODs=Soluble Chemical Oxygen Demand, VFA=Volatile Fatty Acids, Prot=Protein, perCODsbyt=percentage of CODt converted into CODs, Carbo=Carbohydrates, Temp=Temperature, TS=Total Solids, pH).

**Supplementary Figure 3:**

| **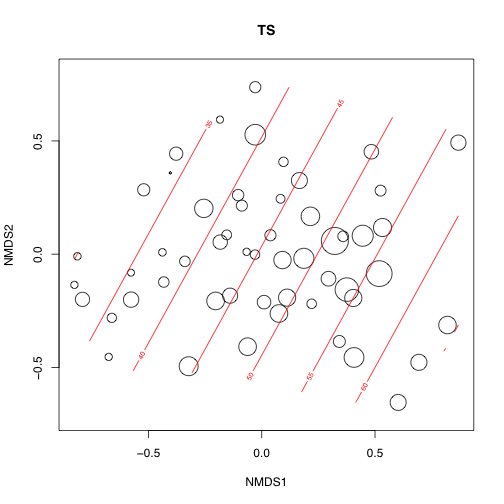** | **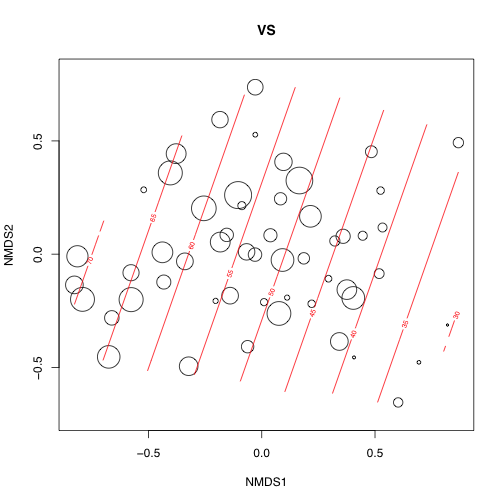** |
| --- | --- |
| **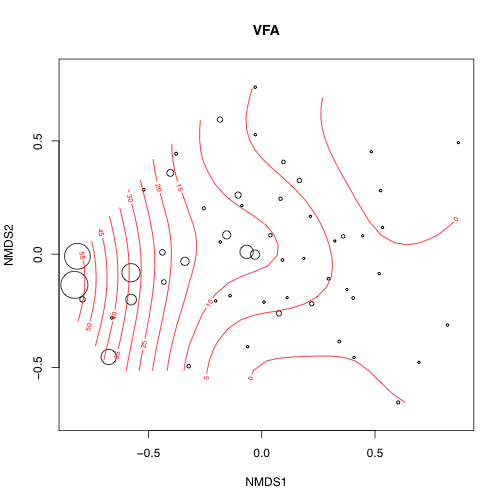** | **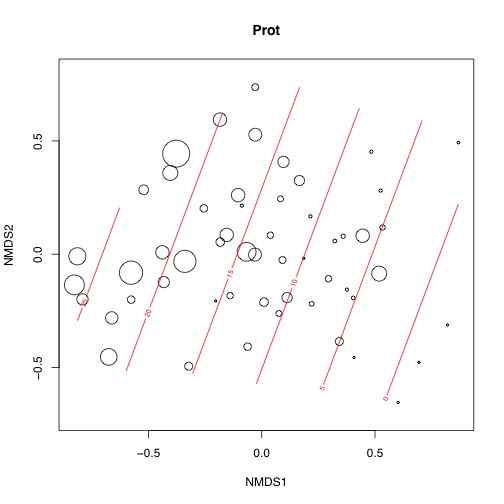** |

**Supplementary Figure 3:** NMDS of family compositions of Vietnamese latrine samples with bubble plots and gradients for the four environmental variables judged significant in Supplementary Table 7 (Total Solids=TS, Volatile Solids=VS, Volatile Fatty Acids=VFA and Protein=Prot) plotted using the envirosurf function of vegan. The size of the bubble indicates the value of the environmental variable.

**Supplementary Figure 4:**

**
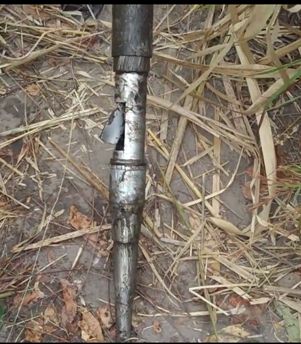

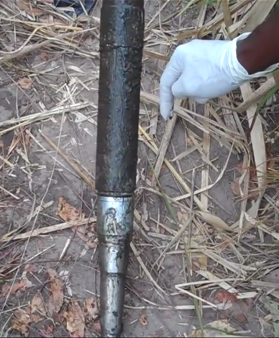
**

**
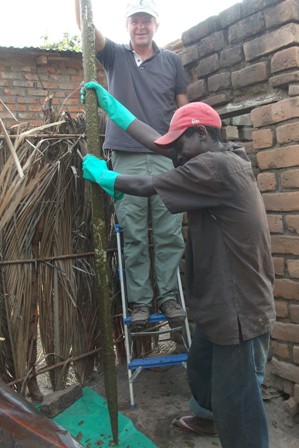
**

**Supplementary Figure 4:** Sampler device used to sample deep latrines with more liquid consistency material. (https://www.youtube.com/watch?v=q5JDu0emYxk)

**Supplementary Table 1**

| Rank | Phylum | Vietnam  % (CI) | Tanzania  % (CI) | Diff. | C. diff |
| --- | --- | --- | --- | --- | --- |
| 1 | Firmicutes* | 32.15(27.3-37.9) | 60.45(47.3-77.2) | 28.3 | 38.3 |
| 2 | Proteobacteria* | 24.78(20.9-29.4) | 4.58(3.3-6.5) | 20.2 | 65.7 |
| 3 | Actinobacteria* | 8.15(6.6-10.0) | 3.14(2.2-4.5) | 5 | 72.5 |
| 4 | Bacteroidetes | 19.81(16.6-23.6) | 15.84(12.0-21.0) | 4 | 77.9 |
| 5 | Synergistetes* | 0.36(0.2-0.5) | 3.23(2.3-4.6) | 2.9 | 81.8 |
| 6 | Deinococcus-Thermus* | 2.80(2.2-3.6) | 0.51(0.3-0.9) | 2.3 | 84.9 |
| 7 | Unknown | 3.37(2.7-4.3) | 5.61(4.0-7.8) | 2.2 | 87.9 |
| 8 | Spirochaetes* | 0.58(0.4-0.8) | 1.93(1.3-2.8) | 1.4 | 89.8 |
| 9 | Chloroflexi | 1.60(1.2-2.1) | 1.45(1.0-2.2) | 1.2 | 91.4 |
| 10 | Tenericutes | 1.59(1.2-2.1) | 0.85(0.5-1.3) | 1.2 | 93 |
| 11 | Verrucomicrobia* | 1.22(0.9-1.6) | 0.13(0.0-0.3) | 1.1 | 94.5 |
| 12 | Planctomycetes | 0.74(0.5-1.0) | 0.30(0.2-0.6) | 0.7 | 95.4 |
| 13 | Fusobacteria | 0.56(0.4-0.8) | 0.19(0.1-0.4) | 0.5 | 96.1 |
| 14 | Acidobacteria | 0.51(0.4-0.7) | 0.57(0.3-0.9) | 0.5 | 96.8 |
| 15 | TM7 | 0.42(0.3-0.6) | 0.00(0.0-1.7) | 0.4 | 97.3 |
| 16 | Fibrobacteres | 0.07(0.0-0.2) | 0.43(0.2-0.8) | 0.4 | 97.8 |
| 17 | Lentisphaerae | 0.31(0.2-0.5) | 0.10(0.0-0.3) | 0.3 | 98.2 |
| 18 | Gemmatimonadetes | 0.25(0.2-0.4) | 0.07(0.0-0.3) | 0.3 | 98.6 |
| 19 | Euryarchaeota | 0.00(0.0-0.7) | 0.19(0.1-0.4) | 0.2 | 98.8 |
| 20 | Cyanobacteria | 0.19(0.1-0.3) | 0.17(0.1-0.4) | 0.2 | 99.1 |
| 21 | Nitrospira | 0.14(0.1-0.3) | 0.00(0.0-1.7) | 0.1 | 99.3 |
| 22 | Thermotogae | 0.00(0.0-0.7) | 0.13(0.0-0.3) | 0.1 | 99.5 |
| 23 | BRC1 | 0.11(0.1-0.2) | 0.07(0.0-0.3) | 0.1 | 99.6 |
| 24 | OD1 | 0.10(0.0-0.2) | 0.00(0.0-1.7) | 0.1 | 99.7 |
| 25 | WS3 | 0.07(0.0-0.2) | 0.00(0.0-1.7) | 0.1 | 99.8 |
| 26 | Chlamydiae | 0.04(0.0-0.1) | 0.00(0.0-1.7) | 0 | 99.9 |
| 27 | OP10 | 0.04(0.0-0.1) | 0.03(0.0-0.2) | 0 | 99.9 |
| 28 | Chrysiogenetes | 0.03(0.0-0.1) | 0.00(0.0-1.7) | 0 | 100 |
| 29 | OP11 | 0.01(0.0-0.1) | 0.00(0.0-1.7) | 0 | 100 |
| 30 | Acidobacteria | 0.51(0.4-0.7) | 0.57(0.3-0.9) | 0 | 100 |

**Supplementary Table 1:** Percentage relative abundance of the 30 phyla in the Dirichlet means in the 55 Vietnamese samples and 24 Tanzanian samples. The upper and lower 95% credible intervals are also given. These are calculated as the maximum posterior estimate (MPE) minus/plus two standard deviations as calculated from the inverse Hessian. Phyla are ranked in order of their contribution to the total mean difference between the two means. This is the second from last column in the table. The total difference was 37%. The cumulative fraction of this difference accounted for by each family is given in the last column of the table.*Indicates those phyla that differ significantly between groups in that their confidence intervals do not overlap. Phyla that were more proportionally abundant in Tanzanian latrines are highlighted in blue, those that were more proportionally abundant in Vietnamese latrines are highlighted in red.

**Supplementary Table 2**

| Rank | Family | Vietnam  %(CI) | Tanzania  %(CI) | Diff. | C. diff |
| --- | --- | --- | --- | --- | --- |
| 1 | Unknown* | 14.27(12.8-15.9) | 27.14(23.2-31.8) | 12.9 | 13.7 |
| 2 | Clostridiaceae* | 3.88(3.3-4.6) | 12.89(10.6-15.6) | 9 | 23.2 |
| 3 | Xanthomonadaceae* | 6.92(6.0-7.9) | 0.51(0.3-0.8) | 6.4 | 30 |
| 4 | Actinomycetales* | 5.32(4.6-6.2) | 1.49(1.0-2.1) | 3.8 | 34.1 |
| 5 | Flavobacteriaceae* | 4.20(3.6-5.0) | 0.40(0.2-0.6) | 3.8 | 38.1 |
| 6 | Ruminococcaceae* | 3.12(2.6-3.8) | 5.87(4.6-7.5) | 2.8 | 41.1 |
| 7 | Synergistaceae* | 0.17(0.1-0.3) | 2.47(1.8-3.4) | 2.3 | 43.5 |
| 8 | Incertae Sedis XI* | 3.02(2.5-3.6) | 5.26(4.1-6.8) | 2.2 | 45.9 |
| 9 | Erysipelotrichaceae* | 1.63(1.3-2.0) | 3.33(2.5-4.5) | 1.7 | 47.7 |
| 10 | Trueperaceae* | 1.87(1.5-2.3) | 0.30(0.2-0.5) | 1.6 | 49.4 |
| 11 | Alcaligenaceae* | 1.68(1.4-2.1) | 0.26(0.2-0.5) | 1.4 | 50.9 |
| 12 | Veillonellaceae* | 0.44(0.3-0.6) | 1.83(1.3-2.6) | 1.4 | 52.4 |
| 13 | Sphingobacteriaceae* | 1.49(1.2-1.9) | 0.09(0.0-0.2) | 1.4 | 53.8 |
| 14 | Burkholderiaceae* | 0.11(0.1-0.2) | 1.50(1.0-2.2) | 1.4 | 55.3 |
| 15 | Syntrophomonadaceae* | 0.24(0.2-0.3) | 1.59(1.1-2.3) | 1.3 | 56.7 |
| 16 | Cryomorphaceae* | 1.51(1.2-1.9) | 0.17(0.1-0.3) | 1.3 | 58.2 |
| 17 | Chitinophagaceae* | 1.48(1.2-1.9) | 0.18(0.1-0.3) | 1.3 | 59.6 |
| 18 | Porphyromonadaceae* | 1.33(1.1-1.7) | 2.61(1.9-3.6) | 1.3 | 60.9 |
| 19 | Pseudomonadaceae* | 1.52(1.2-1.9) | 0.36(0.2-0.6) | 1.2 | 62.1 |
| 20 | Spirochaetaceae* | 0.26(0.2-0.4) | 1.32(0.9-1.9) | 1.1 | 63.3 |
| 21 | Gracilibacteraceae* | 0.10(0.1-0.2) | 1.13(0.8-1.7) | 1 | 64.4 |
| 22 | Phyllobacteriaceae* | 1.20(0.9-1.5) | 0.27(0.2-0.5) | 0.9 | 65.3 |
| 23 | Rhodobacteraceae* | 1.34(1.1-1.7) | 0.46(0.3-0.7) | 0.9 | 66.3 |
| 24 | Peptostreptococcaceae | 4.47(3.8-5.2) | 3.94(3.0-5.2) | 0.8 | 67.2 |
| 25 | Hyphomicrobiaceae* | 0.91(0.7-1.2) | 0.17(0.1-0.3) | 0.7 | 67.9 |
| 26 | Idiomarinaceae* | 0.78(0.6-1.0) | 0.06(0.0-0.2) | 0.7 | 68.7 |
| 27 | Anaerolineaceae* | 0.13(0.1-0.2) | 0.86(0.6-1.3) | 0.7 | 69.5 |
| 28 | Saprospiraceae* | 0.75(0.6-1.0) | 0.06(0.0-0.2) | 0.7 | 70.2 |
| 29 | Halomonadaceae* | 0.80(0.6-1.0) | 0.11(0.1-0.3) | 0.7 | 71 |
| 30 | Alteromonadaceae* | 0.64(0.5-0.8) | 0.00(0.0-1.0) | 0.6 | 71.6 |

**Supplementary Table 2:** Percentage relative abundance of the first 30 out of 180 families in the Dirichlet means in the 55 Vietnamese samples and 24 Tanzanian samples. The upper and lower 95% credible intervals are also given. These are calculated as the maximum posterior estimate (MPE) minus/plus two standard deviations as calculated from the inverse Hessian. Families are ranked in order of their contribution to the total mean difference between the two means. This is the second from last column in the table. The total difference was 37%. The cumulative fraction of this difference accounted for by each family is given in the last column of the table.*Indicates those families that differ significantly between groups in that their confidence intervals do not overlap. Families that were more proportionally abundant in Tanzanian latrines are highlighted in blue, those that were more proportionally abundant in Vietnamese latrines are highlighted in red.

**Supplementary Table 3**

| Rank | OTU | Taxonomic classification | Vietnam  %(CI) | Tanzania  %(CI) | Diff. | C. diff |
| --- | --- | --- | --- | --- | --- | --- |
| 1 | C9140 | Bacteria;Bacteroidetes;Bacteroidia;Bacteroidales;Rikenellaceae;vadinBC27 wastewater-sludge group | 0.02(0.0-0.0) | 3.51(2.9-4.2) | 3.5 | 2.7 |
| 2 | C1168 | Bacteria;Firmicutes;Clostridia;Clostridiales;Clostridiaceae;Clostridium (Clostridiaceae) | 0.35(0.3-0.4) | 3.49(2.9-4.2) | 3.1 | 5.1 |
| 3 | C1161 | Bacteria;Firmicutes;Clostridia;Clostridiales;Clostridiaceae;Clostridium (Clostridiaceae) | 2.09(1.8-2.4) | 4.12(3.4-4.9) | 2.0 | 6.7 |
| 4 | C499 | Bacteria;Firmicutes;Clostridia;Clostridiales;Clostridiaceae | 0.14(0.1-0.2) | 1.84(1.4-2.4) | 1.7 | 8.0 |
| 5 | C3008 | Bacteria;Proteobacteria;Gammaproteobacteria;Xanthomonadales;Xanthomonadaceae;Ignatzschineria | 1.01(0.8-1.2) | 0.09(0.1-0.2) | -0.9 | 8.7 |
| 6 | C4799 | Bacteria;Firmicutes;Clostridia;Clostridiales;Family XI Incertae Sedis (Clostridiales);Sedimentibacter | 0.01(0.0-0.0) | 0.92(0.7-1.2) | 0.9 | 9.4 |
| 7 | C63 | Bacteria;Firmicutes;Clostridia;Clostridiales;Family XI Incertae Sedis (Clostridiales);Gallicola | 0.03(0.0-0.1) | 0.88(0.6-1.2) | 0.9 | 10.1 |
| 8 | C22 | Bacteria;Proteobacteria;Betaproteobacteria;Burkholderiales;Burkholderiaceae;Burkholderia | 0.03(0.0-0.1) | 0.82(0.6-1.1) | 0.8 | 10.7 |
| 9 | C8292 | Bacteria;Firmicutes;Clostridia;Clostridiales;Family XI Incertae Sedis (Clostridiales) | 0.02(0.0-0.0) | 0.77(0.6-1.1) | 0.8 | 11.2 |
| 10 | C3501 | Bacteria;Proteobacteria;Gammaproteobacteria;Xanthomonadales;Xanthomonadaceae | 0.70(0.6-0.9) | 0.02(0.0-0.1) | -0.7 | 11.8 |

**Supplementary Table 3:** Percentage relative abundance of the first 10 out of 12,335 3% OTUs in the Dirichlet means in the 55 Vietnamese samples and 24 Tanzanian samples. The upper and lower 95% credible intervals are also given. These are calculated as the maximum posterior estimate (MPE) minus/plus two standard deviations as calculated from the inverse Hessian. OTUs are ranked in order of their contribution to the total mean difference between the two means. This is the second from last column in the table. The total difference was 129%. The cumulative fraction of this difference accounted for by each family is given in the last column of the table.*Indicates those families that differ significantly between groups in that their confidence intervals do not overlap. OTUs that were more proportionally abundant in Tanzanian latrines are highlighted in blue, those that were more proportionally abundant in Vietnamese latrines are highlighted in red.

**Supplementary Table 4**

| Variable | Df | SumsOfSqs | MeanSqs | F.Model | R2 | Pr(>F) |
| --- | --- | --- | --- | --- | --- | --- |
| Country | 1 | 1.3178 | 1.31781 | 23.4718 | 0.19650 | 0.001 *** |
| Latrine | 26 | 2.5294 | 0.09728 | 1.7327 | 0.37715 | 0.006 ** |
| Depth | 1 | 0.0522 | 0.05216 | 0.9290 | 0.00778 | 0.371 |
| Residuals | 50 | 2.8072 | 0.05614 |  | 0.41858 |  |
| Total | 78 | 6.7066 |  |  | 1.00000 |  |

**Supplementary Table 4:** Permutational multivariate analysis of variance using Bray-Curtis distances for the phylum latrine composition data as a function of country of origin and latrine identity (adonis function vegan - Oksanen et al. 2012). Signif. codes: *** < 0.001‚** < 0.01,* < 0.05‚ . < 0.1.

**Supplementary Table 5**

| Variable | Df | SumsOfSqs | MeanSqs | F.Model | R2 | Pr(>F) |
| --- | --- | --- | --- | --- | --- | --- |
| Country | 1 | 2.9822 | 2.98218 | 37.467 | 0.21915 | 0.001 *** |
| Latrine | 26 | 6.4026 | 0.24625 | 3.094 | 0.47050 | 0.001 *** |
| Depth | 1 | 0.2434 | 0.24342 | 3.058 | 0.01789 | 0.009 ** |
| Residuals | 50 | 3.9797 | 0.07959 |  | 0.29246 |  |
| Total | 78 | 13.6079 |  |  | 1.00000 |  |

**Supplementary Table 5:** Permutational multivariate analysis of variance using Bray-Curtis distances for the family latrine composition data as a function of country of origin and latrine identity (adonis function vegan - Oksanen et al. 2012). Signif. codes: *** < 0.001‚** < 0.01,* < 0.05‚ . < 0.1.

**Supplementary Table 6**

| Variable | Df | SumsOfSqs | MeanSqs | F.Model | R2 | Pr(>F) |
| --- | --- | --- | --- | --- | --- | --- |
| Country | 1 | 4.1797 | 4.1797 | 22.1226 | 0.15395 | 0.001 *** |
| Latrine | 26 | 13.0667 | 0.5026 | 2.6600 | 0.48127 | 0.001 *** |
| Depth | 1 | 0.4575 | 0.4575 | 2.4213 | 0.01685 | 0.001 *** |
| Residuals | 50 | 9.4467 | 0.1889 |  | 0.34794 |  |
| Total | 78 | 27.1506 |  |  | 1.00000 |  |

**Supplementary Table 6:** Permutational multivariate analysis of variance using Bray-Curtis distances for the 3% OTU latrine composition data as a function of country of origin and latrine identity (adonis function vegan - Oksanen et al. 2012). Signif. codes: *** < 0.001‚** < 0.01,* < 0.05‚ . < 0.1.

**Supplementary Table 7**

| Variable | Df | SumsOfSqs | MeanSqs | F.Model | R2 | Pr(>F) |
| --- | --- | --- | --- | --- | --- | --- |
| pH | 1 | 0.2757 | 0.27568 | 3.7534 | 0.02123 | 0.002 ** |
| Temp | 1 | 1.5788 | 1.57876 | 21.4953 | 0.12159 | 0.001 *** |
| TS | 1 | 0.9556 | 0.95562 | 13.0110 | 0.07360 | 0.001 *** |
| VS | 1 | 0.4960 | 0.49602 | 6.7534 | 0.03820 | 0.001 *** |
| VFA | 1 | 0.4894 | 0.48943 | 6.6638 | 0.03770 | 0.001 *** |
| CODt | 1 | 0.1201 | 0.12010 | 1.6352 | 0.00925 | 0.107 |
| CODs | 1 | 0.1254 | 0.12543 | 1.7078 | 0.00966 | 0.115 |
| perCODsbyt | 1 | 0.1402 | 0.14023 | 1.9093 | 0.01080 | 0.059 . |
| NH4 | 1 | 0.3006 | 0.30060 | 4.0928 | 0.02315 | 0.003 ** |
| Prot | 1 | 0.2630 | 0.26299 | 3.5807 | 0.02025 | 0.006 ** |
| Carbo | 1 | 0.5745 | 0.57446 | 7.8214 | 0.04424 | 0.001 *** |
| Country | 1 | 0.7804 | 0.78040 | 10.6254 | 0.06010 | 0.001 *** |
| Latrine | 26 | 4.2435 | 0.16321 | 2.2222 | 0.32683 | 0.001 *** |
| Depth | 1 | 0.0701 | 0.07011 | 0.9546 | 0.00540 | 0.454 |
| Residuals | 35 | 2.5706 | 0.07345 |  | 0.19799 |  |
| Total | 74 | 12.9840 |  |  | 1.00000 |  |

**Supplementary Table 7:** Permutational multivariate analysis of variance using Bray-Curtis distances for latrine composition family data as a function of intrinsic environmental variables, country of origin and latrine identity (adonis function vegan - Oksanen et al. 2012). Signif. codes: *** < 0.001‚** < 0.01,* < 0.05‚ . < 0.1.

**Supplementary Table 8**

| Variable | Df | SumsOfSqs | MeanSqs | F.Model | R2 | Pr(>F) |
| --- | --- | --- | --- | --- | --- | --- |
| pH | 1 | 0.1814 | 0.18139 | 1.5436 | 0.02312 | 0.145 |
| Temp | 1 | 0.1222 | 0.12220 | 1.0399 | 0.01557 | 0.412 |
| TS | 1 | 0.8080 | 0.80804 | 6.8763 | 0.10297 | 0.001 *** |
| VS | 1 | 0.3847 | 0.38473 | 3.2740 | 0.04903 | 0.003 ** |
| VFA | 1 | 0.4660 | 0.46605 | 3.9660 | 0.05939 | 0.001 *** |
| CODt | 1 | 0.1406 | 0.14065 | 1.1969 | 0.01792 | 0.253 |
| CODs | 1 | 0.0816 | 0.08158 | 0.6943 | 0.01040 | 0.695 |
| perCODsbyt | 1 | 0.1759 | 0.17586 | 1.4965 | 0.02241 | 0.150 |
| NH4 | 1 | 0.1390 | 0.13905 | 1.1833 | 0.01772 | 0.287 |
| Prot | 1 | 0.2715 | 0.27152 | 2.3106 | 0.03460 | 0.020 * |
| Carbo | 1 | 0.1405 | 0.14051 | 1.1957 | 0.01791 | 0.289 |
| Residuals | 42 | 4.9354 | 0.11751 |  | 0.62896 |  |
| Total | 53 | 7.8470 |  |  | 1.00000 |  |

**Supplementary Table 8:** Permutational multivariate analysis of variance using Bray-Curtis distances for Vietnam latrine microbial composition family data as a function of intrinsic environmental variables (adonis function vegan - Oksanen et al. 2012). Signif. codes: *** < 0.001‚** < 0.01,* < 0.05‚ . < 0.1.

**Supplementary Table 9**

| Variable | Df | SumsOfSqs | MeanSqs | F.Model | R2 | Pr(>F) |
| --- | --- | --- | --- | --- | --- | --- |
| pH | 1 | 0.13332 | 0.13332 | 1.7650 | 0.05469 | 0.166 |
| Temp | 1 | 0.12631 | 0.12631 | 1.6722 | 0.05182 | 0.170 |
| TS | 1 | 0.47630 | 0.47630 | 6.3057 | 0.19540 | 0.006 ** |
| VS | 1 | 0.09614 | 0.09614 | 1.2728 | 0.03944 | 0.267 |
| VFA | 1 | 0.18765 | 0.18765 | 2.4843 | 0.07698 | 0.077 . |
| CODt | 1 | 0.05669 | 0.05669 | 0.7505 | 0.02326 | 0.571 |
| CODs | 1 | 0.19063 | 0.19063 | 2.5237 | 0.07820 | 0.062 . |
| perCODsbyt | 1 | 0.22261 | 0.22261 | 2.9471 | 0.09132 | 0.037 * |
| NH4 | 1 | 0.09005 | 0.09005 | 1.1922 | 0.03694 | 0.269 |
| Prot | 1 | 0.06846 | 0.06846 | 0.9064 | 0.02809 | 0.471 |
| Carbo | 1 | 0.10963 | 0.10963 | 1.4513 | 0.04497 | 0.204 |
| Residuals | 9 | 0.67981 | 0.07553 |  | 0.27889 |  |
| Total | 20 | 2.43758 |  |  | 1.00000 |  |

**Supplementary Table 9:** Permutational multivariate analysis of variance using Bray-Curtis distances for Tanzanian latrine microbial composition family data as a function of intrinsic environmental variables (adonis function vegan - Oksanen et al. 2012). Signif. codes: *** < 0.001‚** < 0.01,* < 0.05‚ . < 0.1.
